# Evaluation Method of Multiobjective Functions' Combination and Its Application in Hydrological Model Evaluation

**DOI:** 10.1155/2020/8594727

**Published:** 2020-03-10

**Authors:** Jiuyuan Huo, Liqun Liu

**Affiliations:** ^1^School of Electronic and Information Engineering, Lanzhou Jiaotong University, Lanzhou 730070, China; ^2^College of Information Science and Technology, Gansu Agricultural University, Lanzhou 730070, China

## Abstract

Parameter optimization of a hydrological model is intrinsically a high dimensional, nonlinear, multivariable, combinatorial optimization problem which involves a set of different objectives. Currently, the assessment of optimization results for the hydrological model is usually made through calculations and comparisons of objective function values of simulated and observed variables. Thus, the proper selection of objective functions' combination for model parameter optimization has an important impact on the hydrological forecasting. There exist various objective functions, and how to analyze and evaluate the objective function combinations for selecting the optimal parameters has not been studied in depth. Therefore, to select the proper objective function combination which can balance the trade-off among various design objectives and achieve the overall best benefit, a simple and convenient framework for the comparison of the influence of different objective function combinations on the optimization results is urgently needed. In this paper, various objective functions related to parameters optimization of hydrological models were collected from the literature and constructed to nine combinations. Then, a selection and evaluation framework of objective functions is proposed for hydrological model parameter optimization, in which a multiobjective artificial bee colony algorithm named RMOABC is employed to optimize the hydrological model and obtain the Pareto optimal solutions. The parameter optimization problem of the Xinanjiang hydrological model was taken as the application case for long-term runoff prediction in the Heihe River basin. Finally, the technique for order preference by similarity to ideal solution (TOPSIS) based on the entropy theory is adapted to sort the Pareto optimal solutions to compare these combinations of objective functions and obtain the comprehensive optimal objective functions' combination. The experiments results demonstrate that the combination 2 of objective functions can provide more comprehensive and reliable dominant options (i.e., parameter sets) for practical hydrological forecasting in the study area. The entropy-based method has been proved that it is effective to analyze and evaluate the performance of different combinations of objective functions and can provide more comprehensive and impersonal decision support for hydrological forecasting.

## 1. Introduction

The parameters optimization of hydrological models has always been an important research content in the hydrological field, and it has a crucial impact on the overall performance of hydrological models and the reliable results of hydrological forecasting [1]. The essence of hydrological model parameters optimization is that adjusting the parameters of the hydrological model to address the errors and uncertainties in the watershed model simulation so that the model output values are as close as possible to the actual values [2, 3].

The behavior and performance evaluation of the hydrological model is commonly made and reported through comparisons of objective function values of simulated and observed variables. Based on the comparison results, the hydrologist can make mathematical subjective and/or objective estimates of the “closeness” of simulated behavior of the model to observations made within the watershed [4].

The single objective function which can only consider one aspect of hydrological features is adopted in the traditional optimization methods for hydrological model parameter [5, 6]. The engineering practices show that parameter optimization of the hydrological model is intrinsically a high dimensional, nonlinear, multivariable, combinatorial optimization problem which involves a set of different objectives (multiobjective) [7]. To fully excavate the various hydrological feature information contained in hydrological data, multiobjective research has become one of the important directions in hydrological model calibration [8].

Two or more conflicting objectives often have to be simultaneously optimized and the trade-off decisions should be taken between these objectives in the multiobjective optimization [9, 10]. The multiobjective optimization model such as the Pareto theory [11] can reduce the degree of simplification of the model and can more fully reflect the requirements of engineering design, thus providing a decision basis for obtaining the optimal solution set.

Parameters optimization of hydrological model is a typical unconstrained multiobjective optimization problem which determines the model parameters' values by the optimization method to make the selected multiple objective functions achieve maximum or minimum simultaneously. Assuming that all objective functions need to be minimized, the multiobjective optimization problem of hydrological model parameters is expressed in the following equation [12]:

(1) $$\begin{array}{c} f\left(X\right)=\min\left\{f_{1}\left(X\right),f_{2}\left(X\right),\ldots ,f_{N}\left(X\right)\right\},\;X=\left[x_{1},x_{2},\ldots ,x_{D}\right], \end{array}$$

where *f*_*i*_(*X*),  *i*=(1,2,…, *N*) is the *i*-th objective function; *N* is the number of the objective functions; *X* is a vector composed of decision variables, namely, the parameters combination of the hydrological model; and *D* is the number of hydrological model parameters to be optimized. The optimization result is the noninferior parameter combination sets in which feasible regions are limited by the values' ranges of the model parameters. Some researchers have applied the multiobjective method for the parameters optimization problem of hydrological models and achieved good results [13].

In studies and literature of hydrologic modeling, there are a large number of objective functions such as the Nash–Sutcliffe efficiency coefficient (NSE), root mean square error (RMSE), and coefficient of determination (*R*^2^) that are frequently used. Because the objective functions are mutually constrained and each objective function places different emphasis on different systematic or dynamic behavioral errors, it is difficult for a hydrologist to clearly evaluate the advantages and disadvantages of the solutions of parameters optimization.

Most of the related research studies focus on the design of multiobjective algorithm and construction of objective functions, which provides important theoretical basis and practical experience for multiobjective optimization of hydrological model parameters. However, they do not provide much guidance for the selection of the actual objective function combination. Thus, how to study the influence of different objective function combinations on the optimization results of hydrological model parameters and make decisions on these Pareto solution sets has become one of the most important issues.

The technique for order preference by similarity to ideal solution (TOPSIS) which was first proposed by Hwang and Yoon is a well-known method for solving a multiple criteria decision-making problem. TOPSIS is designed based upon the concept that the chosen alternative should have the shortest distance from the positive ideal solution (PIS) and the farthest from the negative ideal solution (NIS) [14]. Thus, it can be adapted to evaluate and give the ranking order of all alternatives in solving a multiple attribute decision-making (MADM) problem. Because of its significant features of comprehensiveness, ease of programming, and reliability, the TOPSIS has been adopted in the decision-making of multiobjective optimization by computing the closeness of the different alternatives to an ideal solution [15].

In the state-of-the-art optimization algorithms, the artificial bee colony (ABC) algorithm [16] which was proposed by Karaboga in 2005 has the advantages of fast convergence speed, strong robustness, and so on. And it has been widely applied in different fields [17, 18]. In this paper, various objective functions related to parameters optimization of hydrological models were collected from the literature and constructed to nine combinations. Then, a multiobjective artificial bee colony algorithm named RMOABC which adopts the mechanisms of the regulation operator and adaptive grid is employed to optimize the hydrological model and obtain the Pareto optimal solution [19]. The parameter optimization problem of the Xinanjiang hydrological model was taken as the application case for long-term runoff prediction in the Heihe River basin. Finally, the TOPSIS method based on the entropy theory is adapted to sort the Pareto optimal solutions to compare the combinations of objective functions and obtain the comprehensive optimal combination.

The paper is organized as follows. The related literatures are reviewed in Section 2.  Section 3 describes the hydrological Xinanjiang model, the study area, the RMOABC algorithm, and the TOPSIS method.  Section 4 summarizes the objective functions and their combinations commonly used in the literature for hydrological model parameters optimization. The selection and evaluation framework of objective functions for hydrological model parameter optimization is proposed in Section 5. The experiment settings, results, and corresponding analyses are discussed in Section 6, and finally, the conclusions are drawn in Section 7.

## 2. Literature Review

In recent years, the ABC algorithm's advantages of great accuracy and satisfactory convergence speed make it suitable for solving the multiobjective optimization problems. Hedayatzadeh et al. designed a multiobjective artificial bee colony (MOABC) based on the Pareto theory and *ε*-domination notion [20]. The performance of a Pareto-based MOABC algorithm has been investigated by Akbari et al. on CEC'09 datasets [21], and the experimental results show that compared with the other multiobjective algorithms, the variants of multiobjective ABC can find solutions with competitive convergence and diversity within a shorter period of time.

The parameter calibration or optimization for the hydrological models has entered the era of multiobjective optimization, and a lot of literatures focus on the multiobjective research studies [22–24]. For the ABC algorithm, a novel multiobjective evolutionary algorithm named multiobjective artificial bee colony (MOABC) algorithm is presented and applied in long-term cascaded hydropower system dispatch in [25]. Pérez et al. presented a multiobjective artificial bee colony-based optimization approach to optimize the allocation of monitoring stations in the design of water quality monitoring networks in river basins [26].

In these studies, a number of objective functions were commonly used for a variety of model calibrations in hydrology such as mean squared error, absolute mean/maximum error, residual bias, Nash objective function [22], the sum of square errors [27], and so on. To evaluate and compare the objective functions, some scholars have also made some efforts. For instance, nine different efficiency criteria for the evaluation of model performance are described and compared through three examples involving an observed streamflow hydrograph from the Wilde Gera catchment in Germany [4]. And Guo et al. presented a method for the comparison of the influence of different objective function combinations on the optimization results of daily streamflow forecasting by a hydrological model [28].

In the evaluation and ranking methods, the TOPSIS is a well-known and widely used method for solving the multiple attribute decision-making problems. For example, Lia et al. extended the concept of TOPSIS to develop a methodology for solving a multiple objective decision-making problem [29]. Abo-Sinna et al. presented a method based on TOPSIS for selection of the large-scale multiobjective nonlinear programming problems with block angular structure [30]. Baky and Abo-Sinna extended the TOPSIS approach to solve bilevel MODM problems [31]. An iterative clustering around latent variable (CLV) based objective entropy weighted TOPSIS approach is proposed for benchmarking building energy performance in a multifactor manner [32]. And a multiobjective mathematical model under several constraints is developed to attain multiple goals simultaneously for sustainable energy investment planning [33].

The TOPSIS method is also adapted and applied for the multiobjective optimization problems (MOPs). For instance, Liu et al. proposed a new method of water distribution system design based on the multiobjective optimization algorithm, and the TOPSIS was used to rank the Pareto optimal solutions [34]. An intuitionistic fuzzy hybrid TOPSIS approach is proposed to handle risk assessment and is applied as a case study on a gas refinery for the welding and lamination task [35]. The concept of TOPSIS was extended to develop a methodology for solving multilevel nonlinear multiobjective decision-making (MLN-MODM) problems of maximization type [36]. TOPSIS was also used to select the optimal cutting conditions for trimming carbon fiber composite that could generate minimum tool wear, surface roughness, and tool temperature simultaneously while maintaining high production rates [15].

For the multiobjective parameter calibration of hydrological models, the TOPSIS has also been adopted by some scholars. A method which combines a genetic algorithm with TOPSIS for the Xinanjiang model is presented to handle the multicriteria parameter calibration problem with a single procedure [37]. A MOPSO algorithm and an entropy-based TOPSIS ranking method were employed to calibrate and verify the Xinanjiang model in Misai catchment [38].

In summary, as the hydrological model and the multiobjective optimization method are determined, the selection and combination of the objective functions become one of the most important factors affecting the prediction quality of the hydrological model. And the research studies on this field are still in the initial stage. Therefore, this paper mainly focuses on collecting the objective functions of hydrological models' optimization from the related literature, optimizing the parameters of the Xinanjiang hydrological model for river runoff forecasting in the Heihe River basin by the RMOABC algorithm and then evaluating and ranking the obtained nondominated solutions based on the TOPSIS method. It would provide a reference method and help for the selection and the combination of objective functions for parameters calibration of hydrological models.

## 3. Study Materials and Methods

### 3.1. Hydrological Model and Study Area

In this paper, a modified Xinanjiang hydrological model [39] with two sources, surface runoff and underground runoff, was selected as the hydrological model. The Xinanjiang model is a conceptual rainfall-runoff (RR) model proposed by Renjun Zhao in 1973 and has been widely used in the humid and semihumid areas in China [40]. The model has ten parameters which have been proved to simulate the runoff well in the working practices [41], including the potential evapotranspiration ratio to pan evaporation *K*; the percentage of impervious in the catchment IMP; the exponential parameter with a single parabolic curve *B*, which represents the spatial distribution nonuniformity of the soil moisture storage capacity over the catchment; the average storage capacity of soil moisture of the upper layer WUM (unit: mm); the average storage capacity of soil moisture of the lower layer WLM (unit: mm); the average storage capacity of soil moisture of the deep layer (unit: mm); the coefficient of soil evaporation in the lower and deep layer of the river basin *C*; the steady infiltration rate FC (unit: mm/hour); the outflow coefficients of the free water storage to groundwater relationships KKG; and the confluence parameters of surface runoff Kr. The value ranges of the ten parameters are shown in Table 1. The detailed description of the modified Xinanjiang model can be found in bib41[7, 12, 41].

The Heihe River watershed in Northwest China was taken as the study area, and parameter optimization for runoff prediction which is a common concern in hydrology researches was taken as the case study. The observation data of daily rainfall, surface evaporation, and runoff from January 1, 1990, to December 31, 1994, were selected for the experiments [7, 12]. The period from January 1, 1990, to December 31, 1990, was chosen as the calibration period. The details of the data can be found in our previous papers [7, 12, 42].

### 3.2. The RMOABC Algorithm

The RMOABC algorithm is proposed to extend the original ABC algorithm based on Pareto theory [43] for handling the multiobjective optimization problems [19]. The regulation operator mechanism is integrated to balance the local search and global search in the evolution process, and the adaptive grid [44] mechanism proposed in the PAES (Pareto archive evolutionary strategy) algorithm was utilized in the RMOABC to produce well-distributed nondominated Pareto solution set in the external archive. Each nondominated solution can be mapped in a certain location in the grid according to the values of multiobjective functions. The grid can adaptively maintain the distribution of candidate solutions stored in the external archive in a uniform way in the evolution process. The details of the RMOABC algorithm and how to integrate the algorithm with the Xinanjiang model can be found in the literature [12, 19].

### 3.3. TOPSIS Method

The technique for order preference by similarity to ideal solution (TOPSIS) was proposed by Hwang et al. in 1981, and it is a useful technique in dealing with multiattribute or multicriteria decision-making problems in the real world [22]. It is based on the concept that the chosen alternative should have the shortest distance to the positive ideal solution (PIS) and the furthest distance from the negative ideal solution (NIS). The positive ideal solution consists of the best performance values among any other alternatives for each attribute, whereas the negative ideal solution is the combination of the worst performance measures [45, 46]. The TOPSIS method can produce a clear preference order of a set of competing designs, which can help decision makers carry out analysis, comparison, and ranking of the alternatives for the problems needed to be solved [22].

For the multiobjective optimization problems, TOPSIS can transfer the *m*-objective (criteria) problem, which are conflicting and difficult in comparison, into a two-objective (the shortest distance from the PIS and the longest distance from the NIS) problem. Then, the biobjective problem can be solved by calculating the relative closeness (overall performance coefficient) to the ideal solution [47]. Thus, TOPSIS can prioritize the nondominated solutions, rank the compromise solutions, and provide decision support for the researchers.

## 4. Multiobjective Functions for Calibration of Hydrological Model

The objective of model calibration is to select the model parameters so that the model simulates the hydrological behavior of the catchment as closely as possible. Objective functions which are defined as functions that must be minimized or maximized in the process of model parameter calibration are commonly used by hydrologists to provide a mathematical estimate of the “closeness” of the simulated behavior to the observed measurements [48]. Each objective function may place different emphasis on different types of simulated and observed behaviors; thus, the selection of the combination of the objective functions is crucial to the optimal parameter result and has been a challenge for the hydrologists.

In this section, we summarized the objective functions and their combinations commonly used in the literature for hydrological model parameters optimization.

### 4.1. Objective Functions in Literature [49]

The Nash–Sutcliffe efficiency coefficient (NSE) [50] and its logarithmic form (LNNSE) [49] are the two common objective functions originating from statistical theory as the primary objectives for model calibration. The two objective functions are defined in equations (2) and (3), respectively. The NSE and the LNNSE functions are a pair of conflicting objective functions [7, 12], where the NSE function evaluates the ability to reproduce all stream flows, but it is known as biased to predict peak flows. On the contrary, the LNNSE function emphasizes low flows.

(2) $$\begin{array}{c} \text{NSE}=1-\frac{\sum_{t=1}^{N}{\left(Q_{\text{sim},t}-Q_{\text{obs},t}\right)}^{2}}{\sum_{t=1}^{N}{\left(Q_{\text{obs},t}-\bar{Q_{\text{obs}}}\right)}^{2}}, \end{array}$$

(3) $$\begin{array}{c} \text{LNNSE}=1-\frac{\sum_{t=1}^{N}{\left(\ln\left(Q_{\text{sim},t}\right)-\ln\left(Q_{\text{obs},t}\right)\right)}^{2}}{\sum_{t=1}^{N}{\left(\ln\left(Q_{\text{sim},t}\right)-\bar{\ln\left(Q_{\text{obs}}\right)}\right)}^{2}}, \end{array}$$

where *N* is the total number of time steps in the calibration period, *Q*_sim*,t*_ is the simulated runoff data value at time *t*, *Q*_obs*,t*_ is the observed data value at time *t*, and $\bar{Q_{\text{obs}}}$ is the average of the observation data. The two objective functions are optimized to the maximum at the same time in the parameters optimization process. The larger value means the better performance of the model forecasting.

### 4.2. Objective Functions in Literature [51]

Madsen applied the following numerical performance statistics to measure the different calibration objectives:

1. Overall volume error: a good agreement between the average simulated and observed catchment runoff volume (i.e. a good water balance).

(4) $$\begin{array}{c} E_{\text{RMOV}}=\frac{1}{N}\left|\overset{N}{\underset{t=1}{\sum }}\left(Q_{\text{obs},t}-Q_{\text{sim},t}\right)\right|. \end{array}$$

(2) Overall root mean square error (RMSE): a good overall agreement of the shape of hydrograph.

(5) $$\begin{array}{c} E_{\text{RMS}}=\sqrt{\frac{1}{N}\overset{N}{\underset{t=1}{\sum }}{\left(Q_{\text{obs},t}-Q_{\text{sim},t}\right)}^{2}}. \end{array}$$

(3) Average RMSE of peak flow events: a good agreement of the peak flows with respect to timing, rate, and volume.

(6) $$\begin{array}{c} E_{\text{RMS}\_H1}=\frac{1}{M_{\text{p}}}\sum_{j=1}^{M_{\text{p}}}\sqrt{\frac{1}{n_{j}}\sum_{t=1}^{n_{j}}{\left(Q_{\text{obs},t}-Q_{\text{sim},t}\right)}^{2}}. \end{array}$$

(4) Average RMSE of low flow events: a good agreement for low flows.

(7) $$\begin{array}{c} E_{\text{RMS}\_L1}=\frac{1}{M_{\text{l}}}\overset{M_{l}}{\underset{j=1}{\sum }}\sqrt{\frac{1}{n_{k}}\sum_{t=1}^{n_{k}}{\left(Q_{\text{obs},t}-Q_{\text{sim},t}\right)}^{2}.} \end{array}$$

In Equations (4)–(7), *N* is the total number of time steps in the calibration period; *Q*_obs*,t*_ is the observed discharge data value at time *t*; *Q*_sim*,t*_ is the simulated runoff data value at time *t*; *M*_p_ is the number of peak flow events; *M*_l_ is the number of low flow events; *n*_*j*_ is the number of time steps in peak flow event *j*; and *n*_*k*_ is the number of time steps in low flow event *k*. Peak flow events are defined as periods where the observed discharge is above a given threshold level. Similarly, low flow events are defined as periods where the observed discharge is below a given threshold. The peak flow events were defined as periods with flow above a threshold value of 0.75 m^3^/s, and low flow events were defined as periods with flow below a threshold value of 0.2 m^3^/s.

### 4.3. Objective Functions in Literature [52]

In literature [52], the mean squared logarithmic error *E*_MSL_ and the mean fourth-power error *E*_*M*4_ of measured flow and forecasted flow were selected as the objective functions. The two objective functions are defined in equations (8) and (9):

(8) $$\begin{array}{c} E_{\text{MSL}}=\frac{1}{N}\sum_{t=1}^{N}{\left(\ln\left(Q_{\text{sim},t}\right)-\ln\left(Q_{\text{obs},t}\right)\right)}^{2}, \end{array}$$

(9) $$\begin{array}{c} E_{M4}=\frac{1}{N}\sum_{t=1}^{N}{\left(Q_{\text{sim},t}-Q_{\text{obs},t}\right)}^{4}, \end{array}$$

where *N* is the total number of time steps in the calibration period, *Q*_sim*,t*_ is the simulated runoff data value at time *t*, and *Q*_obs*,t*_ is the observed data value at time *t*.

Due to the logarithmic operation, *E*_MSL_ makes the fitting error of low flow more significant, so it is more focused on the fitting of low flow value. For the impact of the fourth power, the fitting error of high flow value has a greater contribution; thus, *E*_*M*4_ is more focused on the fit of high flow values. The experimental results showed that there is a typical noninferior relationship between the objective functions *E*_MSL_ and *E*_*M*4_.

### 4.4. Objective Functions in Literature [53]

The parameters of the ANN-based monthly streamflow forecasting model are estimated by several multiobjective optimization algorithms with three pairs of objective functions. (1) mean squared logarithmic error (*E*_MSL_) vs. mean squared derivative error (*E*_MSD_), (2) mean fourth-power error (*E*_*M*4_) vs. mean squared derivative error (*E*_MSD_), and (3) mean squared logarithmic error (*E*_MSL_) vs. mean fourth-power error (*E*_*M*4_). The definition of *E*_MSD_ objective function is shown in the following equation:

(10) $$\begin{array}{c} E_{\text{MSD}}=\frac{1}{N-1}\sum_{t=2}^{N}{\left(\left(Q_{\text{obs},t}-Q_{\text{obs},t-1}\right)-\left(Q_{\text{sim},t}-Q_{\text{sim},t-1}\right)\right)}^{2}, \end{array}$$

where *N* is the total number of time steps in the calibration period, *Q*_sim*,t*_ is the simulated runoff data value at time *t*, and *Q*_obs*,t*_ is the observed data value at time *t*.

The *E*_MSL_ function is more suitable for low flows due to the logarithmic transformation. *E*_*M*4_ is considered to be an indicator of the goodness of fit for high flows because it amplifies the effect of the deviation. The experiments demonstrated that *E*_MSD_ can be taken as an indicator of the fit of the shape of the hydrograph, and it should be used in combination with *E*_MSL_ or *E*_*M*4_.

### 4.5. Objective Functions in Literature [3]

The Nash–Sutcliffe efficiency coefficient (NSE) and the overall runoff relative error *R*_*E*_ were selected as the objective functions in [3]. *R*_*E*_ is defined in the following equation:

(11) $$\begin{array}{c} R_{E}=1-\frac{\sum_{t=1}^{N}Q_{\text{sim},t}}{\sum_{t=1}^{N}Q_{\text{obs},t}}, \end{array}$$

where *N* is the total number of time steps in the calibration period, *Q*_sim*,t*_ is the simulated runoff data value at time *t*, and *Q*_obs*,t*_ is the observed data value at time *t*.

### 4.6. Objective Functions in Literature [54]

There are four metrics used in [54]—two common statistical metrics that emphasize high and low flows and two hydrological metrics that emphasize the water balance and the midrange flow regime. These metrics capture four different but important components of the hydrograph while allowing for a comparison between statistical and hydrological metrics.

The first statistical metric is the commonly used root mean squared error (*E*_RMS_) which has been defined in equation (5), and it emphasizes fitting the high flow portions of the hydrograph.

The second statistical metric is transformed root mean squared error *E*_TRMS_. The simulated and observed flow time series are first transformed by a Box–Cox transformation (equation (12) with a *λ* value of 0.3, which has a similar effect as a log transformation. *E*_TRMS_ emphasizes low flow portions of the hydrograph and is defined in equation (13).

(12) $$\begin{array}{c} Z=\frac{{\left(1+Q\right)}^{\lambda }-1}{\lambda }, \end{array}$$

(13) $$\begin{array}{c} E_{\text{TRMS}}=\sqrt{\frac{1}{N}\sum_{t=1}^{N}{\left(Z_{\text{obs},t}-Z_{\text{sim},t}\right)}^{2}}, \end{array}$$

where *N* is the total number of time steps in the calibration period, *Z*_sim,*t*_ is the transformed simulated runoff data value at time *t*, and *Z*_obs,*t*_ is the observed data value at the time. After the Box‐Cox transformation, the observed and simulated flow data can help the parameter estimation method to better adapt to the low flows.

The third metric is the runoff coefficient error (*E*_ROC_) which captures the overall accuracy of the water balance by first combining the flows into one characteristic hydrological descriptor. It is defined in the following equation:

(14) $$\begin{array}{c} E_{\text{ROC}}=\left|\frac{\bar{Q_{\text{sim, annual}}}-\bar{Q_{\text{obs, annual}}}}{\bar{P_{\text{obs, annual}}}}\right|, \end{array}$$

where $\bar{Q_{\text{sim,annual}}}$ and $\bar{Q_{\text{obs,annual}}}$ are the simulated and observed mean annual runoff volume and $\bar{P_{\text{obs,annual}}}$ is the mean annual precipitation which is 388.95 mm/year in the research area.

The final metric is the slope of the flow duration curve error (*E*_SFDC_), which measures how well the model captures the distribution of midlevel flows. The slope of a watershed's flow duration curve indicates the variability, or flashiness, of its flow magnitudes. *E*_SFDC_ metric is calculated by equation (15), and it is simply the absolute error in the slope of the flow duration curve between the 30 and 70 percentile flows.

(15) $$\begin{array}{c} E_{\text{SFDC}}=\left|\frac{\left(Q_{\text{sim},70}-Q_{\text{sim},30}\right)-\left(Q_{\text{obs}70}-Q_{\text{obs},30}\right)}{40}\right|, \end{array}$$

where *Q*_sim,30_ and *Q*_sim,70_ are the 30 and 70 percentile flows of the simulated flow duration curve and *Q*_obs,30_ and *Q*_obs70_ are the 30 and 70 percentile flows of the observed flow duration curve.

### 4.7. Objective Functions in Literature [55]

The performance measures used in [55] are the Nash–Sutcliffe efficiency coefficient (NSE), the coefficient of determination for linear regression (*R*^2^), and percent bias (*P*_BIAS_). The coefficient of determination for linear regression is a measure of how well the regression line represents the data. The coefficient of determination *R*^2^ is defined as the squared value of the coefficient of correlation which is defined in the following equation:

(16) $$\begin{array}{c} R^{2}={\left(\frac{\sum_{t=1}^{N}\left(Q_{\text{obs},t}-\overset{¯}{Q_{\text{obs}}}\right)\left(O_{\text{sim},t}-\bar{O_{\text{sim}}}\right)}{\sqrt{\sum_{t=1}^{N}{\left(Q_{\text{obs},t}-\bar{Q_{\text{obs}}}\right)}^{2}}\sqrt{\sum_{t=1}^{N}{\left(Q_{\text{sim},t}-\bar{Q_{\text{sim}}}\right)}^{2}}}\right)}^{2}, \end{array}$$

where *N* is the total number of time steps in the calibration period, *Q*_sim,*t*_ is the simulated runoff data value at time *t*, *Q*_obs,*t*_ is the observed data value at time *t*, $\bar{Q_{\text{sim}}}$ is the average of the simulated data, and $\bar{Q_{\text{obs}}}$ is the average of the observation data. The range of *R*^2^ lies between 0 and 1 which describes how much of the observed values can be reproduced by the prediction. A bigger value means better prediction.

*P*_BIAS_ which is defined in equation (17) measures the average tendency (deviation) of the simulated data to their observed counterparts and is expressed as a percentage. The optimal value of *P*_BIAS_ is 0.0, with low-magnitude values indicating accurate model simulation [56].

(17) $$\begin{array}{c} P_{\text{BIAS}}=\frac{\left|\sum_{t=1}^{N}\left(Q_{\text{obs},t}-Q_{\text{sim},t}\right)\right|}{\sum_{t=1}^{N}Q_{\text{obs},t}}\times 100. \end{array}$$

### 4.8. Objective Functions in Literature [8]

Three evaluation functions are selected as the objective functions for the runoff prediction of the hydrological model: the root mean square error  *E*_MSR_ which represents the overall simulation result, the water volume balance function *E*_RMOV_, and the mean squared logarithmic error *E*_MSL_ (overall volume error). *E*_MSR_ is defined in the following equation:

(18) $$\begin{array}{c} E_{\text{MSR}}=\frac{1}{N}\sum_{t=1}^{N}\left(\sqrt{Q_{\text{sim},t}}-\sqrt{Q_{\text{obs},t}}\right){}^{2}. \end{array}$$

### 4.9. Objective Functions in Literature [28]

As shown in equations (19)–(24), six objective functions were constructed based on the absolute error of the model forecasted flow and the measured flow to establish three objective function combinations, and a simple method for the comparison of the influence of different objective function combinations was presented in [28]. The objective function combinations include *E*_RMS_*L*_ vs. *E*_RMS_*H*_, *R*_*L*_^2^ vs. *R*_*H*_^2^, and *E*_MA_*L*_ vs. *E*_MA_*H*_. Among them, the subscripts *H* and *L* denote the flood peak flow objective and the low flow objective, respectively.

(19) $$\begin{array}{c} E_{\text{RMS}\_L2}=\sqrt{\frac{\sum_{i=1}^{M_{\text{l}}}\sum_{t=1}^{n_{k}}{\left(Q_{\text{obs},t}-Q_{\text{sim},t}\right)}^{2}}{\sum_{i=1}^{M_{\text{l}}}n_{k}}}, \end{array}$$

(20) $$\begin{array}{c} E_{\text{RMS}\_H2}=\sqrt{\frac{\sum_{i=1}^{M_{\text{p}}}\sum_{t=1}^{n_{j}}{\left(Q_{\text{obs},t}-Q_{\text{sim},t}\right)}^{2}}{\sum_{i=1}^{M_{\text{p}}}n_{j}}}, \end{array}$$

(21) $$\begin{array}{c} R_{L}^{2}=\frac{1}{M_{\text{l}}}\overset{M_{\text{l}}}{\underset{j=1}{\sum }}\frac{\sum_{t=1}^{n_{k}}{\left(Q_{\text{obs},t}-Q_{\text{sim},t}\right)}^{2}}{\sum_{t=1}^{n_{k}}{\left(Q_{\text{obs},t}-\bar{Q_{\text{obs},t}}\right)}^{2}}, \end{array}$$

(22) $$\begin{array}{c} R_{H}^{2}=\frac{1}{M_{\text{p}}}\overset{M_{\text{lp}}}{\underset{j=1}{\sum }}\frac{\sum_{t=1}^{n_{j}}{\left(Q_{\text{obs},t}-Q_{\text{sim},t}\right)}^{2}}{\sum_{t=1}^{n_{j}}{\left(Q_{\text{obs},t}-\bar{Q_{\text{obs},t}}\right)}^{2}}, \end{array}$$

(23) $$\begin{array}{c} E_{\text{MA}\_L}=\frac{\sum_{i=1}^{M_{\text{l}}}\sum_{t=1}^{n_{k}}\left|Q_{\text{obs},t}-Q_{\text{sim},t}\right|}{\sum_{i=1}^{M_{\text{l}}}n_{k}}, \end{array}$$

(24) $$\begin{array}{c} E_{\text{MA}\_H}=\frac{\sum_{i=1}^{M_{\text{p}}}\sum_{t=1}^{n_{j}}\left|Q_{\text{obs},t}-Q_{\text{sim},t}\right|}{\sum_{i=1}^{M_{\text{p}}}n_{j}}, \end{array}$$

where *N* is the total number of time steps in the calibration period; *Q*_obs*,t*_ is the observed discharge data value at time *t*; *Q*_sim,*t*_ is the simulated runoff data value at time *t*; $\bar{Q_{\text{obs}}}$ is the average of the observation data; *M*_p_ is the number of peak flow events; *M*_l_ is the number of low flow events; *n*_*j*_ is the number of time steps in peak flow event *j*; and *n*_*k*_ is the number of time steps in low flow event *k*.

Peak flow events or low flow events are defined as periods where the observed discharge is above or below a given threshold level, respectively. Similar to Section 4.2, the low flow threshold is 0.2 m^3^/s and the peak flow threshold is 0.75 m^3^/s. In the literature [28], the researchers found that the combination of *E*_RMS_*L*2_, *E*_RMS_*H*2_, and *E*_MA_*L*_ can effectively balance the multiple objectives and can provide more comprehensive decision-making support for the forecasters.

## 5. Selection and Evaluation Framework of Objective Function Combination

Solving a multiobjective optimization problem of parameter calibration for the hydrological model is to search for the nondominated solutions that can be expressed in terms of Pareto theory in the objective space of parameters. However, for the hydrologist, the nondominated solutions should be evaluated and ranked, and a set of parameters (i.e., one of the nondominated solutions) should be selected from the Pareto set for the application of the hydrological model. Thus, after the multiobjective optimization algorithm calibrating the parameters of a hydrological model and obtaining a set of nondominated solutions, the task of how to evaluate and rank these solutions corresponding to the objective function combination and select the optimal solution that meets the needs of decision makers has become more important. In this paper, a widely used ranking method called technique for order preference by similarity to ideal solution (TOPSIS) was used to prioritize the nondominated solutions and help the hydrologists find the appropriate combination of objective functions.

### 5.1. Entropy-Based TOPSIS

TOPSIS approach was originally proposed for the comprehensive evaluation of multiple scenarios in the case of multiple attributes. It is one of the best-known ranking methods of alternatives for MADM problems and can be easily adapted to different fields. If the evaluation alternative is closest to the ideal solution and far away from the negative ideal solution, it is the best alternative; otherwise, it is the worst [46]. For the multiobjective parameters optimization problem of the hydrological model in this paper, each optimization objective function represents an attribute. Thus, the nondominated solutions generated from RMOABC algorithm were treated as alternatives and the objectives were taken as attributes.

In this paper, a TOPSIS method is employed to rank the nondominated solutions, and the entropy theory of information is also adapted to assign objective weights reasonably for different attributes. The entropy-based TOPSIS procedure consists of the following steps [32, 38]:

1. Calculate the decision matrix *D*.
2. The decision matrix *D* is constructed according to the Pareto optimal solution set. Assume that there are *n* Pareto optimal solutions (alternatives) *A*_*i*_ (*i* = 1, 2,…, *n*) to be evaluated against *m* criteria (objective functions) *C*_*j*_ (*j* = 1, 2,…, *m*). Then, as shown in equation (25), the decision matrix *D* contains performance ratings for the *i*-th alternative (Pareto solution) *A*_*i*_ with respect to the *j*-th criteria (objective function) *C*_*j*_, which are denoted as *x*_*ij*_.

(25) $$\begin{array}{c} D=\begin{array}{c} A_{1}\\ A_{2}\\ \vdots \\ A_{i}\\ \vdots \\ A_{n} \end{array}\overset{\begin{array}{cccccc} C_{1} & C_{2} & \ldots & C_{j} & \ldots & C_{m} \end{array}}{\left[\begin{array}{cccccc} x_{11} & x_{12} & \ldots & x_{1j} & \ldots & x_{1m}\\ x_{21} & x_{22} & \ldots & x_{2j} & \ldots & x_{2m}\\ \vdots & \vdots & \vdots & \vdots & \vdots & \vdots \\ x_{i1} & x_{i2} & \ldots & x_{ij} & \ldots & x_{im}\\ \vdots & \vdots & \vdots & \vdots & \vdots & \vdots \\ x_{n1} & x_{n2} & \ldots & x_{nj} & \ldots & x_{nm} \end{array}\right]}. \end{array}$$

  In general, the evaluation criteria are classified into two types: benefit and cost. For criteria of benefit, larger value means more valuable, while for the criteria of cost criteria, smaller value means more valuable.
(2) Construct normalized decision matrix *R*.
  Then, the above decision matrix *D* is normalized and trended to obtain a normalized matrix *R.* According to the criteria type of benefit or cost, the normalized value *r*_*ij*_ can be calculated as follows:

(26) $$\begin{array}{c} r_{ij}=\left\{\begin{array}{cc} \frac{x_{ij}-x_{j}^{\min}}{x_{j}^{\max}-x_{j}^{\min}}, & \text{for benefit criteria,}\\ \frac{x_{j}^{\max}-x_{ij}}{x_{j}^{\max}-x_{j}^{\min}}, & \text{for cost criteria,} \end{array}\right. \end{array}$$

  where *x*_*j*_^max^=∨_*i*=1_^*n*^*x*_*ij*_ and *x*_*j*_^min^=∧_*i*=1_^*n*^*x*_*ij*_ are the maximum and the minimum values of the *j*-th criteria among *n* alternatives and *r*_*ij*_ represents the normalized performance of *A*_*i*_ with respect to attribute *C*_*j*_. After the normalized transformation, the normalized decision-making matrix is represented as follows:

(27) $$\begin{array}{c} R={\left(r_{ij}\right)}_{n\times m}, \end{array}$$

  where 0 ≤ *r*_*ij*_ ≤ 1 which means the optimal value for each attribute is 1 and the worst value is 0. This step can transform various attribute dimensions into nondimensional attributes to allow comparisons across criteria.
(3) Calculate the weighted normalized decision matrix.
  Considering the different importance of each criterion, the weighted normalized decision matrix *Z* can be constructed as follows:

(28) $$\begin{array}{c} Z={\left(z_{ij}\right)}_{n\times m}={\left(w_{j}r_{ij}\right)}_{n\times m}, \end{array}$$

  where *w*_*j*_ is the weight of the *j*-th criterion, *w*_*j*_ > 0, and ∑_*j*=1_^*m*^*w*_*j*_=1.
  Generally, the weights of attributes are determined from the experience of decision maker depending on the specific problem. For more accuracy, a reasonable method based on the theory of information entropy for assigning objective weights of attributes is adapted to calculate objective weights [38]. According to the definition of entropy, the entropy value of each attribute (objective) *j* can be measured as follows:

(29) $$\begin{array}{c} H_{j}=-k\sum_{i=1}^{n}y_{ij}\ln y_{ij}, \end{array}$$

  where *H*_*j*_ is the entropy value of objective *j*, *k* is a constant (*k*=1/ln(*n*)), *n* is the amount of nondominated solutions, and *y*_*ij*_ is defined as

(30) $$\begin{array}{c} y_{ij}=\frac{\left(1+x_{ij}\right)}{\sum_{i=1}^{n}\left(1+x_{ij}\right)}, \end{array}$$

  where *x*_*ij*_ is the *i*-th nondominated solution (alternative) under the *j*-th criteria (objective function). Thus, if the amount of objectives is *m*, the weight for each criterion *j* is given by the following equation:

(31) $$\begin{array}{c} w_{j}=\frac{\left(1-H_{j}\right)}{\sum_{k=1}^{m}\left(1-H_{k}\right)}. \end{array}$$

(4) Determine the positive ideal solution (best) and negative ideal solution (worst).
  The positive ideal solution (PIS), whose index consists of the best value for each criterion, is defined in equation (32). The negative ideal solution (NIS), whose index consists of the worst value for each criterion, is defined in equation (33).

(32) $$\begin{array}{c} A^{+}=\left\{\vee_{i=1}^{n}z_{ij}\right\}=\left\{z_{1}^{+},z_{2}^{+},\ldots ,z_{j}^{+},\ldots ,z_{m}^{+}\right\}, \end{array}$$

(33) $$\begin{array}{c} A^{-}=\left\{\wedge_{i=1}^{n}z_{ij}\right\}=\left\{z_{1}^{-},z_{2}^{-},\ldots ,z_{j}^{-},\ldots ,z_{m}^{-}\right\}. \end{array}$$

(5) Calculate the separation measures by the *m*-dimensional Euclidean distance for each alternative.
  The separation from the positive ideal alternative can be expressed as equation (34) and similarly, the separation from the negative ideal alternative can be expressed as equation (35).

(34) $$\begin{array}{c} d_{i}^{+}=\sqrt{\sum_{j=1}^{m}{\left(z_{ij}-z_{j}^{+}\right)}^{2}},\;i=1,2,\ldots ,n, \end{array}$$

(35) $$\begin{array}{c} d_{i}^{-}=\sqrt{\sum_{j=1}^{m}{\left(z_{ij}-z_{j}^{-}\right)}^{2}},\;i=1,2,\ldots ,n. \end{array}$$

(6) Calculate the relative closeness (overall performance coefficient) to the ideal solution.
  Thus, considering the entropy-TOPSIS, the relative closeness coefficient of each nondominated solution is expressed as follows:

(36) $$\begin{array}{c} C_{i}^{*}=\frac{d_{i}^{-}}{d_{i}^{+}+d_{i}^{-}}, \end{array}$$

  where *i*=1,2,…, *n* and 0 ≤ *C*_*i*_^*∗*^ ≤ 1.
(7) Rank the preference order.
  Finally, by sorting the closeness coefficients *C*_*i*_^*∗*^ calculated in Step 6, the ranking order of all alternatives can be obtained. According to the concept of the TOPSIS method, the higher value of *C*_*i*_^*∗*^ means that it has higher priority, and on the contrary, the smaller value of *C*_*i*_^*∗*^ means it has lower priority. Thus, the alternative with the highest *C*_*i*_^*∗*^ value will be the optimal alternative.

### 5.2. Combinations of Objective Functions

In this section,we summarized and collated the objective functions and their combinations from the relevant literatures on hydrological model parameter optimization. Information on the combination of objective functions, related descriptions, objective function characteristics, and related literature is summarized in Table 2. The hydrological model parameter optimization experiments will be carried out according to these combinations of objective functions, and the runoff prediction of the hydrological model will be carried out. We have summarized a total of 19 objective functions and 9 objective function combinations whose number has been denoted from 1 to 9.

There are several reasons why we do not take all the object functions as the combination. (1) All objective functions are researched and summarized by many scientists in hydrological fields based on domain knowledge and practical work experience. It cannot be replaced and modified at will. (2) Because there are 19 kinds of objective functions, multiobjective functions cannot be optimized by algorithms at the same time, and an objective solution set that can balance each objective function cannot be obtained. (3) According to the experiments in Section 6.2.1 in the paper, it can be seen that there are strong conflicts in some objective functions, which generate a large number of NAN values and illegal values so that the convergence of all objective functions cannot be achieved.

Since NSE, LNNSE, *R*_*E*_, and *R*^*2*^ are the objective functions for finding the maximum, other objective functions are optimized for the minimum values. Therefore, to unify the types of the extreme value of these objective functions and to easily compare the values of objective functions, these four objective functions have been modified and converted into minimum types which are shown in equations (37)–(40):

(37) $$\begin{array}{c} \text{NSE}{}^{\prime }=\frac{\sum_{t=1}^{N}{\left(Q_{\text{sim,}t}-Q_{\text{obs},t}\right)}^{2}}{\sum_{t=1}^{N}{\left(Q_{\text{obs},t}-\bar{Q_{\text{obs}}}\right)}^{2}}, \end{array}$$

(38) $$\begin{array}{c} \text{LNNSE}{}^{\prime }=\frac{\sum_{t=1}^{N}{\left(\ln\left(Q_{\text{sim},t}\right)-\ln\left(Q_{\text{obs},t}\right)\right)}^{2}}{\sum_{t=1}^{N}{\left(\ln\left(Q_{\text{sim},t}\right)-\bar{\ln\left(Q_{\text{obs}}\right)}\right)}^{2}}, \end{array}$$

(39) $$\begin{array}{c} R_{E}^{\prime }=\left|1-\frac{\sum_{t=1}^{N}Q_{\text{sim},t}}{\sum_{t=1}^{N}Q_{\text{obs},t}}\right|, \end{array}$$

(40) $$\begin{array}{c} R^{2^{\prime }}=1-{\left(\frac{\sum_{t=1}^{N}\left(Q_{\text{obs},t}-\overset{¯}{Q_{\text{obs}}}\right)\left(O_{\text{sim},t}-\overset{¯}{Q_{\text{sim}}}\right)}{\sqrt{\sum_{t=1}^{N}{\left(Q_{\text{obs},t}-\overset{¯}{Q_{\text{obs}}}\right)}^{2}}\sqrt{\sum_{t=1}^{N}{\left(Q_{\text{sim},t}-\overset{¯}{Q_{\text{sim}}}\right)}^{2}}}\right)}^{2}. \end{array}$$

### 5.3. Selection and Evaluation Framework

**Figure 1:**
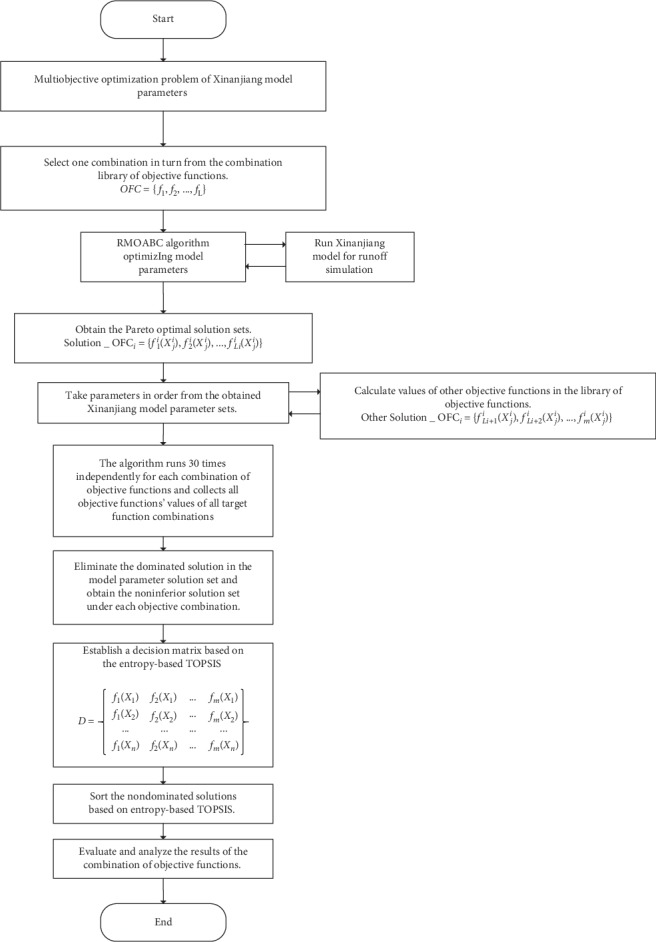
Selection and evaluation framework of combinations of objective functions.

To comprehensively evaluate these objective functions and their combinations which are summarized in Table 2, we constructed a simple selection and evaluation framework of objective functions which is shown in Figure 1.

Firstly, a library of objective function combinations for hydrological model parameter optimization is established. Assuming that there are *K* kinds of combinations of objective functions, the library can be shown in the following equation:

(41) $$\begin{array}{c} {\text{OFC}}_{i}=\left\{f_{1}^{i},f_{2}^{i},\ldots ,f_{j}^{i},\ldots ,f_{Li}^{i}\right\}, \end{array}$$

where OFC_*i*_ is the *i*-th combination of objective functions, *i*=1,2,…, *K*, and *j*=1,2,…, *Li* is the number of the objective functions in the *i*-th combination. The objective functions in each combination can be partially identical but cannot be fully identical; thus, *Li* is different for OFC_*i*_.

Then, the RMOABC optimization algorithm is employed to solve the parameter optimization problem of Xinanjiang model one by one for the objective function combination, and the corresponding Pareto optimal solutions (i.e., parameters sets) for Xinanjiang model are obtained. According to the model parameters in the Pareto solution, other remaining objective function values are calculated one by one. Finally, the relative merits of the optimization scheme corresponding to the Pareto solution obtained for each objective function combination are compared using the method of entropy-based TOPSIS.

The selection and evaluation framework of combinations of objective functions is a simple method for comparing the optimization performance of different objectives combination, and the specific steps are described as follows:

1. Select one combination in turn from the combinations library of objective functions which is defined as

(42) $$\begin{array}{c} \text{OFC}=\left\{f_{1},f_{2},\ldots ,f_{L}\right\}. \end{array}$$

(2) Use the RMOABC optimization algorithm to call the Xinanjiang model for simulating the basin runoff of the Heihe River. The noninferior solution sets (optimal model parameters) under all combinations of objective functions can be obtained, as shown in the following equation:
  (43) $$\begin{array}{c} \text{Solution}\_{\text{OFC}}_{i}=\left\{f_{1}^{i}\left(X_{j}^{i}\right),f_{2}^{i}\left(X_{j}^{i}\right),\ldots ,f_{Li}^{i}\left(X_{j}^{i}\right)\right\}, \end{array}$$

where *i* = 1, 2,…, *m*; *j* = 1, 2,…, *ni*; Solution_OFC_*i*_ is the non-inferior solutions set of the *i*-th combination of the objective function; *X*_*j*_^*i*^ is the combination of model parameters corresponding the *j*-th noninferior solution to the *i*-th combination of the objective functions; and *ni* is the number of obtained noninferior solutions at the *i*-th combination of objective function.
(3) Take the parameters set obtained by each combination of objective functions in sequence and set them as the parameter to run the Xinanjiang hydrological model . Then, the values of the rest of the objective functions in the library are calculated. In this way, the values of all the objective functions corresponding to each combination of objective functions can be obtained, as shown in the following equation:
  (44) $$\begin{array}{c} {\text{OtherSolution\_OFC}}_{i}=\left\{f_{Li+1}^{i}\left(X_{j}^{i}\right),f_{Li+2}^{i}\left(X_{j}^{i}\right),\ldots ,f_{m}^{i}\left(X_{j}^{i}\right)\right\}, \end{array}$$

where *i* = 1, 2,…, *m*; *j* = 1, 2,…, *ni*; and OtherSolution_OFC_*i*_ are other objective functions to the *i*-th combination of the objective function.
(4) To eliminate the influence of random factors of the algorithm, the algorithm runs 30 times independently for each combination of objective functions. Then, the noninferior solution sets of each combination of objective functions obtained in 30 runs are combined together.
(5) Collect and organize all the objective function values corresponding to the parameter set of all objective function combinations and eliminate the dominated solutions in the model parameter solution sets to obtain all noninferior solution sets under each combination, as shown in the following equation:
  (45) $$\begin{array}{c} \text{AllSolution}\_{\text{OFC}}_{i}=\left\{f_{1}^{i}\left(X_{j}^{i}\right),f_{L2}^{i}\left(X_{j}^{i}\right),\ldots ,f_{Li}^{i}\left(X_{j}^{i}\right),\ldots ,f_{m}^{i}\left(X_{j}^{i}\right)\right\}, \end{array}$$

  where *i* = 1, 2,…, *m*; *j* = 1, 2,…, *ni*; and AllSolution_OFC_*i*_ is the noninferior solution set obtained by the *i*-th combination of objective functions.
(6) Establish a decision matrix based on the entropy-based TOPSIS method to sort all noninferior solutions of the combination of objective functions.
(7) Comprehensively evaluate the objective function combination. According to the TOPSIS ranking results of the noninferior solution sets obtained in the foregoing operations, quantitatively describe and compare the influence of each combination of objective functions on the parameters optimization of the hydrological model.

## 6. Experiment Analyses

### 6.1. Experiment Preparations

The experiments of the simulation were taken on the multiobjective evolutionary algorithm (MOEA) framework [57] in the paper. The MOEA is a powerful and efficient platform and provides a Java library of open source for developing and evaluating the multiobjective optimization algorithms. The hardware environment of simulation experiments is a PC with 4 cores of Intel i7 2.6 GHz CPU and 8 GB of RAM.

#### 6.1.1. Parameter Settings

Because the selection of algorithm parameters can greatly affect the execution performance, we adopted the recommended parameter settings from the related research studies [12] for the RMOABC algorithm. The swarm size of bees (NP) is set to 100, the external archive capacity is set to 100, and the adaptive grid number is set to 25. For the *Limit* parameter, it is set to 0.25 *∗* NP *∗* *D* [58]. *D* denotes the dimension of decision variables of the optimized problem, that is, the number of Xinanjiang model parameters, 10.

The dimension of the objective functions is set to the number of objective functions in the selected combination. Based on our previous experience [12], for the calibration of the parameters of Xinanjiang hydrological model, a maximum number of model evaluations in the range 2000–4000 normally ensure an efficient calibration. Thus, the stopping criterion for the optimization algorithm is the maximum number of model evaluations, and it is set to 3000.

#### 6.1.2. Evaluation Indicators of Multiobjective Optimization Algorithms

An important issue in multiobjective optimization is to evaluate and compare the quality of solution sets. The straightforward way to compare the quality of solution sets is visualization, but visual comparison cannot quantify the difference between solution sets and also becomes harder with more objectives involved [59]. Thus, quantitative evaluation methods can be interpreted as how well it represents the Pareto front and used to measure the performances of the multiobjective optimization algorithms [28]. In general, the quality indicators of solution set can be divided into four aspects: convergence, spread, uniformity, and cardinality. Convergence of a solution set refers to the closeness of the set to the Pareto front which denotes the distance between the calculated noninferior front and the known true noninferior front or approximate true noninferior front. Spread of a solution set considers the region of the set covering. Uniformity of a set refers to how even the solution distribution is in the set, and an equidistant spacing amongst solutions is desirable. Cardinality of a solution set refers to the number of solutions in the set [59].

According to the literature [59], the most frequently used evaluation indicators were selected to evaluate all the four aspects of performance of the nondominated solution sets in this paper. The generational distance (GD) [60] was selected to evaluate the convergence performance, and the spacing (SP) [61] was selected to evaluate the uniformity performance. The inverted generational distance (IGD) [62] was chosen to evaluate the comprehensive quality of convergence and spread, and the hypervolume (HV) [63] was also chosen to evaluate the comprehensive quality of convergence, spread, and cardinality. Due to the desirable practical usability and theoretical properties, the HV indicator is arguably the most commonly used quality indicator and is suitable for many real-world optimization scenarios. And the HV indicator is the only popular unary indicator having the strict Pareto compliance which implies that only the Pareto front achieves a unique optimal value for a problem [59]. We also added the computational time (Time) which was recorded in seconds to evaluate the execution efficiency of the algorithms.

### 6.2. Experimental Result Analyses

#### 6.2.1. Model Parameter Optimization Result

According to the above experimental scheme and algorithm parameter setting, the RMOABC optimization algorithm is used to optimize the parameters of the Xinanjiang model based on the nine objective function combinations of the hydrological model parameters compiled in Section 5.2. Each group of experiments was carried out 30 times, and the optimal parameter set of each group of experiments was collected together, and the dominant solution in each group was removed. As shown in Table 3, a total of 8,435 optimal parameter solution sets are obtained. The Pareto optimal solutions obtained by the objective function combination 1, 3, 4, 5, and 8 are relatively small. They are 95, 120, 178, 325, and 355, respectively. The objective function combination 6, 7, and 9 obtained more than 1,000 Pareto optimal solutions, and in particular, the objective function combination 2 obtained a total of 3,629.

However, since the nine objective function combinations include 19 objective functions, we take each parameter solution from each objective function combination in turn according to the steps of the selection and evaluation framework and then calculate the corresponding values of the remaining objective functions. For example, in the objective function combination 2, four objective functions are included, and one parameter solution is sequentially extracted from the corresponding 3,629 solutions, and the runoff data are simulated by the Xinanjiang model, and then the remaining 15 objective functions' values are calculated. In this way, we can get all the values of 19 objective functions corresponding to the 8,435 optimal parameter solution sets.

Nevertheless, we found that there are many NAN values in the newly calculated objective function values which are mainly concentrated in the objective functions of LNNSE, *E*_MSL_, *E*_TRMS_, *E*_MSR_, and *E*_MA_*L*_. After analysis, the main reasons for these NAN values are as follows. (1) As there is a value of 0 in the observation data or the prediction data, the natural logarithm calculation in LNNSE and *E*_MSL_ will generate the NAN value. (2) In the hydrological model, 0 is taken as the divisor. The occurrence of the NAN value in the objective function affects the parameter solution which cannot be sorted. Therefore, we removed all the solutions which contain NAN value in the objective function. After elimination of all the solutions containing NAN values, a total of 6,918 remaining multiobjective parameter solutions are obtained.

As described in Section 5.2, in order to facilitate the comparison of these 19 objective functions, we have converted all of these objective functions into minimization functions, and the function values should be in the range [0, 1], and the closer the function value is to 0, the higher the degree of optimization of the solution. However, after reviewing the result set of the objective function values, it was found that a large amount of data larger than 1 exists in the remaining objective function values. It shows that there are strong conflicts between these objective functions. The optimal parameter set obtained for some objective functions does not perform well or even worse on other objective functions. To compare the performance of the objective function, we removed all model parameter solutions containing objective function values greater than 1 from the experimental result set. After eliminating the solutions with NAN value and value greater than 1, a total of 165 optimal parameter solution sets are obtained, and there are about 6.900 solutions whose objective function value exceeds 1.

Next, we compared the Pareto dominance of 19 objective function values with 165 optimal parameter solutions and found that there is no dominant solution in these optimal parameter solutions. Therefore, the nine objective function combinations finally obtained 165 optimal parameter solutions.

**Figure 2:**
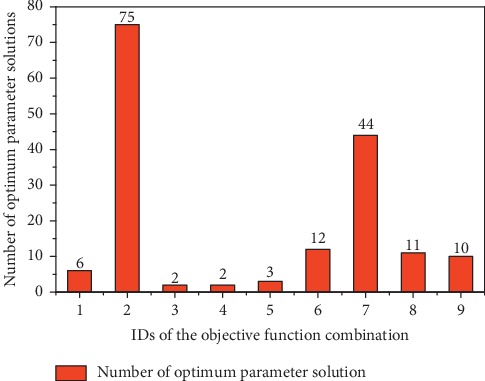
The proportion of the optimal solution set of each objective function combination in the total number of solution sets.

The number of optimal solution sets of each objective function combination in the total number of solution sets is shown in Table 4 and Figure 2. It can be seen that the objective function combination 2 and the objective function 7 obtain more optimal solutions, respectively. Their proportions are 45.45% and 26.67%, respectively. The optimal solutions obtained by the remaining objective combinations are relatively small, especially the objective function combinations 3 and 4 which only obtained two optimal solution sets. The objective function combinations 2 and 7 also obtained 3,629 and 1,667 solution sets before the data preprocessing, respectively, which indicates that these two objective function combinations have stronger ability to obtain the optimal parameter solution sets, and the ability of their obtained parameter solution sets is also strong for adapting to other objectives.

#### 6.2.2. Calculating the Closeness of the Optimal Solution Sets of Objective Function Combinations

After the above preprocessing for the Pareto optimal parameter solutions generated by the RMOABC algorithms based on the 19 objective functions for a validation period, a total of 165 model optimal parameter sets are obtained for the Xinanjiang model. The sample Pareto optimal parameter solutions are shown in Table 5.

The Pareto optimal solution of the hydrological model contains 19 optimization objectives, that is, there are 19 attribute values. Except the optimal solution corresponding to the attribute value of the objective function combination, the remaining objective function values are all calculated by the parameter solution. Then, the 165 attribute value matrices of the nine objective function combinations are combined to form a TOPSIS decision matrix *D* of 165 × 19.

Then, the decision matrix *D* will be solved according to the steps of the entropy-based TOPSIS method described in Section 5.1. In the TOPSIS method, the weight of each objective function is the key factor affecting the ranking result of optimal solutions, but the common method is to assign the corresponding weight values to the objective functions based on the experience of the decision maker for the specific problem. In this paper, 19 objective functions have been selected for the research; therefore, we cannot assign weights to these objective functions reasonably and scientifically. Thus, we adopt the method of calculating the information entropy of each objective function in the optimal solutions to determine their weights for more accuracy [38].

Table 6 indicates the entropy value and weights of 19 functions which were calculated according to equations (29) and (31). From the table, it can be found that the 19 objective functions show unequal weights. *R*_*E*_ function shows the highest weight, and NSE and LNNSE also have an important effect on the model parameters optimization. *E*_ROC_ indicates the lowest weight, 0.00000, which means the objective function almost has no effect on the hydrological model optimization results. And *E*_*M*4_, *E*_MSD_, *E*_SFDC_, and  *E*_MSR_ also have little importance for the optimization problem.

After the completion of the weight calculation for each objection function, the weighted normalized decision matrix *Z* can be calculated. Then, the positive ideal solution (PIS) and negative ideal solution (NIS) will be determined. And the separation measures by the *m*-dimensional Euclidean distance for each alternative will be obtained. Finally, based on the entropy-TOPSIS method, the overall performance coefficient of each nondominated solution which is expressed as relative closeness to the ideal solution will be obtained according to equation (36). Since the number of optimal solutions is large, Table 7 shows all the 19 objective function values of the sample Pareto optimal parameter solutions in Table 5 and their relative closeness which is calculated by the entropy-based TOPSIS method.

#### 6.2.3. Ranking the TOPSIS Preference Order

Finally, by sorting the closeness coefficients *C*_*i*_^*∗*^ in descending order, the ranking order of all alternatives (i.e., Pareto optimal solutions) can be obtained. The higher value of *C*_*i*_^*∗*^ means the higher priority; the smaller value of *C*_*i*_^*∗*^ means the lower priority. The maximum value of the overall ranking of closeness coefficients calculated by the 165 optimal parameter solution sets is 0.95196 which means the solution is the optimal alternative, and the minimum value is 0.25942 which means the related solution is the worse alternative. To compare the optimal solution sets obtained by each objective function combination, we multiply the value of closeness coefficients by 100 and convert it into a 5-point system, that is, the converted value is “Excellent” between 90 and 100, “Good” between the 80 to 89, “Medium” between 70 and 79, “Pass” between 60 and 69, and “Poor” for values lower than 60.

**Figure 3:**
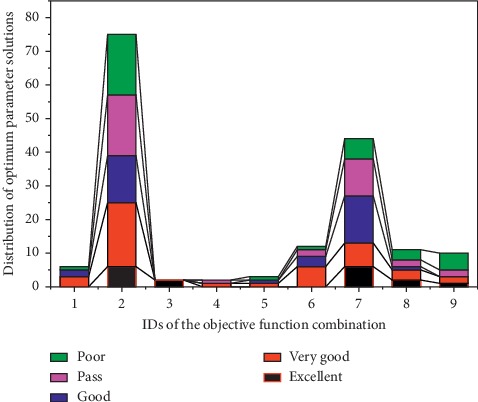
Distribution of the optimal parameter solutions after TOPIS sorting in the 5-segment position interval.

After dividing the ranking score into 5 levels, we then analyzed the distribution of the optimal parameter solution sets obtained by the nine combinations of the objective functions in the 5-segment position interval. The results are shown in Table 8 and Figure 3. We can see that in the 165 optimal solution sets, objective combinations 2 and 7 obtain a larger number of optimal solutions, while other objective combinations obtain fewer optimal solutions. Taking objective combination 1 as an example, five of its six optimal solutions are above the “Pass” level, indicating that the quality of the solution corresponding to the objective combination is higher, but its number of solutions is very small, accounting for only 3.64% of the total solution. It shows that the ability of these objective function combinations to obtain the optimal parameter solution sets is not strong, and the obtained parameter solution set cannot adapt to other objective function requirements which means that they do not have advantages in the competition.

It can be seen from the distribution diagram of TOPSIS sorting position in Figure 3 that the number of distributions of objective combinations 2 and 7 in the five sorting intervals is very uniform, especially in the Excellent and Good intervals, and the solutions of the top ranking are superior in number and ratio to the other objective functions. It shows that objective function combinations 2 and 7 can achieve more and better solutions than other objective combinations.

To compare the quality of the solutions corresponding to each objective combination, we extracted all the solutions that reach the excellent level and sorted them according to the closeness value. A total of 17 Pareto optimal parameter sets have reached the excellent level whose relative closeness values are bigger than 0.90, and the ranking results are shown in Table 9. The solution having the highest TOPSIS relative closeness indicated with an asterisk in the table is the compromise solution which fits the decision maker's preference most.

It can be seen from the table that among the top 10 solutions, the first to fourth and the sixth are the optimal solution sets corresponding to the objective combination 2, and in all 17 solution sets, the objective combination 2 has 6 which accounts for 35.29%. It shows that the optimal solution corresponding to the objective combination 2 not only has higher quality but also has stronger competitiveness and can basically adapt to the goals and requirements of all 19 objective functions. The other outstanding objective function combination, i.e., combination 7, also has 6 solution sets to reach the excellent level, but its corresponding highest order solution is only in the 7th position, and the rest of the solutions are ranked in the lower positions. The solutions related to the remaining objective combinations do not perform very well. For example, the objective combinations 3 and 8 only have 2 solutions, and the objective combination 9 only has 1 solution in the excellent level. Not only the quality of the solution is low, but the number of solutions is also very small. And for the objective combinations 1, 4, 5, and 6, they even do not have the optimal solution set to achieve the “Excellent” level. Therefore, it can be seen from the experimental results that the solutions corresponding to the objective function 2 and the objective function 7 are more widely distributed than the other objective combinations, and their performances are better, especially the solution quality corresponding to the objective function 2 is more excellent.

#### 6.2.4. Comparison of Optimal Parameter Results Corresponding to Each Objective Function Combination

First, each parameter set is named in turn by the sequence number sorted by TOPSIS. Then, according to the ID number of each objective function combination, the solution set with the highest ranking of each target function combination is extracted from the TOPSIS sorted parameter solution set, and the 10 optimized parameter values, the corresponding closeness values, and the corresponding sorting results are shown in Table 10. For example, the objective combination 2 corresponds to solution 1, the objective combination 8 corresponds to solution 5, and so on. Meanwhile, the 19 objective function values for optimal parameter solution related to the 9 combinations of objective functions are listed in Table 11. The solutions of the objective combinations 2, 8, 7, 9, and 3 are at the “Excellent” level, and the solutions corresponding to the remaining objective combinations are at the “Good” level.

**Figure 4:**
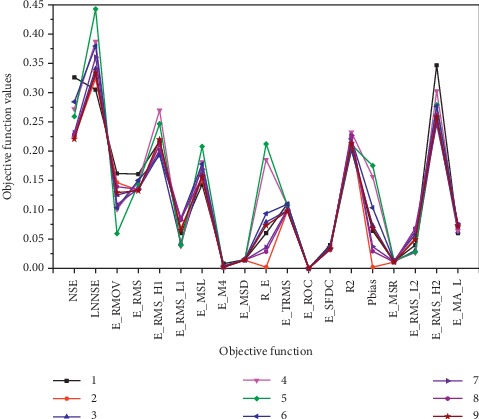
Comparison of the 19 objective function values of the optimal parameter solution related to the 9 combinations of objective functions.

In Section 5.2, we made corresponding modifications to the objective functions and converted the maximum objective function to the minimum type, and the values of each objective function were normalized whose value range is limited in [0, 1]. The smaller the value, the better the solution related to the objective function. Therefore, to compare the relative differences of the attributes of the nine objective function combinations corresponding to the optimal solutions, we compare the 19 objective function values in Table 11, and the comparison results are shown in Figure 4.

It can be seen from the figure that among these optimal solutions, the parameter set of the objective function combinations 2, 8, and 7 performs better, and smaller value can be obtained in each objective function. It shows that the objective combinations can meet the optimization requirements of these 19 objective functions and can achieve better optimization results. Among them, the objective function combination 2 performs particularly well, and *R*_*E*_ and *P*_BIAS_ clearly achieved better results. However, the corresponding solutions of objective combinations 9 and 3 are also in excellent grade, but they have some fluctuations in the objective function values; that is, they perform better in some objective functions but perform poorly in other objective functions. Especially for the solutions corresponding to the objective combinations 1, 6, 4, and 5 in the “Good” level, the fluctuations are more obvious. Taking the objective combination 5 as an example, the optimal optimization effect is obtained on *E*_RMOV_, but it achieved the worst optimization results in LNNSE, *E*_MSL_, *R*_*E*_, and *P*_BIAS_. It is indicated that the solutions related to these objective functions are not stable enough to fully cover the requirements of the 19 objective functions and they do not have strong adaptability.

#### 6.2.5. Result Comparison of Multiobjective Evaluation Indicators

It can be seen from the above experimental results that compared with other objective function combinations, the objective function combinations 2 and 7 can obtain a widely distributed and high-quality parameter solution set. In order to compare the quality of the corresponding solutions of these two objective function combinations, the generational distance (GD), spacing (SP), inverted generational distance (IGD), and Hypervolume (HV) were adopted to evaluate the performance of convergence, uniformity, spread, and cardinality of the solution set. And the computational time (Time) was used to evaluate the execution efficiency of the algorithms.

Firstly, according to the approximate noninferior solution set obtained in the previous experiments and the noninferior solution sets obtained by the 30 independent runs of algorithm, the five quality indicators of noninferior solutions under each objective combination are calculated, and then the performances of the two objective combinations on the optimization of hydrological model parameters can be quantitatively described and compared. The five performance metrics for parameter optimization problem are shown in Table 12. Mean and SD represents the average value and the standard deviation of the experiments results, respectively. The best values among the results for the optimization problem are shown in bold.

From the results of Table 12, the conclusions consistent with the above can be obtained. (1) The optimal noninferior parameter solution sets corresponding to objective combinations 2 and 7 have good performance in convergence, uniformity, spread, and cardinality. (2) In comparison, the optimal parameter solutions of objective function combination of 2 have relatively better results for generational distance (GD), spacing (SP), inverted generational distance (IGD), and hypervolume (HV). It demonstrated that the combination 2 is better than the combination 7 in terms of the performance indicators. (3) Because the number of objective functions in combination 2 is bigger than combination 7, it has to take more execution time to obtain the final solution set. (4) Overall, the objective combinations 2 and 7 can effectively coordinate between the 19 various conflicting objectives which are arranged in the paper, and it is expected to provide forecasters with a more comprehensive basis for decision-making.

## 7. Conclusions

In the multiobjective parameters' optimization process of the hydrological model, a combination of efficiency criteria (objective functions) is defined as the mathematical measures to evaluate how well the model simulation fits the available observed data. There are a large number of objective functions, and each objective function may place more or less emphasis on different aspects of the hydrological model. However, how to choose and evaluate the different combination of objective functions efficiently has been a challenge for even the most experienced hydrologists. To help the researchers select and evaluate the objective functions that are suitable for their research, nine objective function combinations consisting of 19 objective functions were collected from the literature and arranged in this paper.

Then, a selection and evaluation framework of objective function combinations was presented. The daily runoff forecasting model, Xinanjiang model, is taken as an application case, and the RMOABC algorithm is introduced to find the nondominated parameter solutions for handling the optimization problem of the hydrological model in the Heihe River basin. And a simple information entropy-based TOPSIS ranking method is adapted to rank the nondominated parameter solutions and compare the performance of optimization results of different objective function combinations.

From the optimization results, it can be found that there are a large number of conflicts between the objective functions of objective combinations, which leads to the result that optimal parameter solutions are difficult to satisfy the optimization tasks of all objective functions. Therefore, the combination of objective functions should be chosen carefully and reasonably; otherwise, the obtained optimal parameters will not reflect the real characteristics of the hydrological model and thus cannot complete the hydrological model runoff forecasting task well.

Then, the information entropy-based TOPSIS ranking method was taken to calculate the closeness of the optimal solution sets of the objective function combinations, and the ranking order of Pareto optimal solutions was obtained by sorting the closeness coefficients *C*_*i*_^*∗*^ in descending order. Based on the analysis of the optimization results, the number of distributions of the objective combinations 2 and 7 in the five sorting intervals is very uniform, especially in the “Excellent” and “Good” levels. And their Pareto solutions with the highest ranking have a great advantage in the number and proportion of the solution sets to other objective function combinations which shows that objective function combinations 2 and 7 can achieve more and better solutions than other combinations. Then, we compared the optimal parameter results corresponding to each objective function combined with the multiobjective evaluation indicator results. The solution corresponding to objective combination 2 has better performance in the quality indicators.

The experiment results showed that the convergence and distribution of combination 2 of objective functions are better than the other combinations, and it can effectively balance the performance of various objectives. It also showed that the entropy-based TOPSIS can provide an effective and better method to calculate objective weights that permit trade-offs between attributes and rank the nondominated solutions impersonally for the parameters' optimization problem of the hydrological model, and it can also provide a more comprehensive decision basis for hydrological forecasting. The future work is to construct a more accurate and balanced combination of objective functions that has good distribution and convergence in different objective spaces.

## Figures and Tables

**Table 1:** The value range of the modified Xinanjiang model parameters with two sources [41].

Parameter name	*K*	IMP	*B*	WUM	WLM	WDM	*C*	FC	KKG	Kr
Lower limit	0.001	0.001	0.001	5.000	50.000	50.000	0.001	0.001	0.001	0.001
Upper limit	1.000	0.500	1.000	30.000	100.000	200.000	0.300	50.000	0.990	10.000

**Table 2:** The summary of the combinations of objective functions.

No.	Combination	Description	Characteristics	Literatures
1	NSE vs. LNNSE	NSE emphasizes to high flow; LNNSE emphasizes to low flow.	NSE and LNNSE are all benefit type.	[49]
2	*E*_RMOV_ vs. *E*_RMS_ vs. *E*_RMS_*H*1_ vs. *E*_RMS_*L*1_	*E*_RMOV_ is a good agreement for good water balance; *E*_RMS_ emphasizes to high flows; *E*_RMS_*H*1_ emphasizes to high flows; *E*_RMS_*L*1_ emphasizes to low flows.	These four functions are all cost type.	[51]
3	*E*_MSL_ vs. *E*_*M*4_	*E*_MSL_ emphasizes to low flows; *E*_*M*4_ emphasizes to high flows.	*E*_MSL_ and *E*_*M*4_ are all cost type.	[52]
4	*E*_MSL_ vs. *E*_MSD_ vs. *E*_*M*4_	*E*_MSL_ emphasizes to low flow; *E*_*M*4_ emphasizes to high flows; *E*_MSD_ emphasizes to high flows.	*E*_MSL_, *E*_*M*4_ and *E*_MSD_ are all cost type.	[53]
5	NSE vs. *R*_*E*_	*NSE* emphasizes to high flows; *R*_*E*_ emphasizes to low flows.	NSE and *R*_*E*_ are all benefit type.	[3]
6	*E*_RMS_ vs. *E*_TRMS_ vs. *E*_ROC_ vs. *E*_SFDC_	*E*_RMS_ emphasizes to high flows; *E*_TRMS_ emphasizes to low flows; *E*_ROC_ emphasizes to water balance; *E*_SFDC_ emphasizes to midlevel flows.	*E*_RMS_, *E*_TRMS_, *E*_ROC_ and *E*_SFDC_ are all cost type.	[54]
7	NSE vs. *R*^2^ vs. *P*_BIAS_	NSE and *R*^2^ emphasize to high flow; *P*_BIAS_ measures the average tendency of the simulated data to their observed one.	NSE and *R*^2^ are benefit type; *P*_BIAS_ is cost type.	[55]
8	*E*_MSR_ vs. *E*_RMOV_ vs. *E*_MSL_	*E*_MSR_ emphasizes to high flows; *E*_RMOV_ is a good agreement for water balance; *E*_MSL_ emphasizes to low flows.	*E*_MSR_, *E*_RMOV_, and *E*_MSL_ are all cost type.	[8]
9	*E*_RMS_*L*2_ vs. *E*_RMS_*H*2_, vs. *E*_MA_*L*_	*E*_RMS_*H*2_ emphasizes to high flows; *E*_RMS_*L*2_ and *E*_MA_*L*_ emphasize to low flows.	*E*_RMS_*L*2_, *E*_RMS_*H*2_, and *E*_MA_*L*_ are all cost type.	[28]

**Table 3:** A number of optimal solutions in the preprocessing of the objective functions values generated by the RMOABC algorithms for the 9 objective functions' combinations.

No.	Combination	Number of optimal solutions	Number of optimal solutions after removing NAN values	Number of optimal solutions after removing NAN values and illegal values	Number of optimal solutions after removing NAN values, illegal values, and dominated solution
1	NSE vs. LNNSE	95	78	6	6
2	*E*_RMOV_ vs. *E*_RMS_ vs. *E*_RMS_*H*1_ vs. *E*_RMS_*L*1_	3629	3016	75	75
3	*E*_MSL_ vs. *E*_*M*4_	120	88	2	2
4	*E*_MSL_ vs. *E*_MSD_ vs. *E*_*M*4_	178	140	2	2
5	NSE vs. *R*_*E*_	325	262	3	3
6	*E*_RMS_ vs. *E*_TRMS_ vs. *E*_ROC_ vs. *E*_SFDC_	1042	838	12	12
7	NSE vs. *R*^2^ vs. *P*_BIAS_	1667	1496	44	44
8	*E*_MSR_ vs. *E*_RMOV_ vs. *E*_MSL_	355	320	11	11
9	*E*_RMS_*L*2_ vs. *E*_RMS_*H*2_, vs. *E*_MA_*L*_	1024	827	10	10

*Total*		8435	7065	165	165

**Table 4:** The proportion of the optimal solution set of each objective function combination in the total number of solution sets.

ID	Number of optimal solutions	Proportion (%)
1	6	3.64
2	75	45.45
3	2	1.21
4	2	1.21
5	3	1.82
6	12	7.27
7	44	26.67
8	11	6.67
9	10	6.06

**Table 5:** The sample Pareto optimal parameter solutions for the Xinanjiang model after the preprocessing.

ID	*K*	IMP	*B*	WUM	WLM	WDM	*C*	FC	KKG	Kr
1	0.44702	0.24567	0.98926	11.43949	85.16284	143.35409	0.00010	29.63879	0.99000	4.08941
1	0.14949	0.30469	0.20173	30.00000	50.00000	169.39066	0.06320	7.27659	0.99000	5.31799
1	0.24108	0.36977	0.77250	12.54337	75.82264	138.12316	0.03286	42.77099	0.99000	6.00000
2	0.17873	0.29657	0.13358	30.00000	50.00000	200.00000	0.00010	38.98337	0.99000	6.00000
2	0.22421	0.26762	0.60919	16.46257	92.43665	76.03592	0.08534	14.96132	0.99000	5.04023
…	…	…	…	…	…	…	…	…	…	…

**Table 6:** Entropy values and weights of the 19 objective functions.

Entropy	NSE	LNNSE	*E*_RMOV_	*E*_RMS_	*E*_RMS_*H*1_	*E*_RMS_*L*1_	*E*_MSL_	*E*_*M*4_	*E*_MSD_	*R*_*E*_	*E*_TRMS_	*E*_ROC_	*E*_SFDC_	*R* ^2^	*P*_*BIAS*_	*E*_MSR_	*E*_*R*MS_*L*2_	*E*_RMS_*H*2_	*E*_MA_*L*_
Value	0.998567	0.998681	0.999868	0.999912	0.999456	0.999915	0.999553	0.999986	0.999996	0.998493	0.999960	1.000000	0.999997	0.999101	0.999116	0.999996	0.999934	0.999399	0.999780
Weight	0.17278	0.15911	0.01596	0.01067	0.06558	0.01029	0.05391	0.00172	0.00052	0.18177	0.00482	0.00000	0.00035	0.10848	0.10663	0.00043	0.00794	0.07248	0.02657

**Table 7:** The sample objective function values of Pareto optimal parameter solutions and their closeness calculated by the entropy-based TOPSIS method.

ID	19 objectives																			Closeness
	NSE	LNNSE	*E*_RMOV_	*E*_RMS_	*E*_RMS_*H*1_	*E*_RMS_*L*1_	*E*_MSL_	*E*_*M*4_	*E*_MSD_	*R*_*E*_	*E*_TRMS_	*E*_ROC_	*E*_SFDC_	*R* ^2^	*P*_BIAS_	*E*_MSR_	*E*_RMS_*L*2_	*E*_RMS_*H*2_	*E*_MA_*L*_	
1	0.74614	0.99416	0.00000	0.24361	0.33585	0.08971	0.46653	0.03892	0.02914	0.02606	0.16232	0.00002	0.04791	0.24335	0.02676	0.02881	0.05790	0.55631	0.10976	0.52341
1	0.36291	0.63628	0.02681	0.16989	0.21261	0.05884	0.29859	0.00732	0.01667	0.00522	0.12159	0.00000	0.03949	0.19458	0.00519	0.01720	0.04725	0.33807	0.08345	0.77118
1	0.51656	0.48720	0.04673	0.20269	0.29084	0.07522	0.22863	0.01645	0.01812	0.10516	0.13564	0.00011	0.04131	0.18954	0.11752	0.01778	0.04847	0.44453	0.07274	0.73661
2	0.86420	0.96255	0.00183	0.26217	0.51969	0.09751	0.45170	0.04922	0.01876	0.03494	0.19023	0.00003	0.02246	0.86061	0.03376	0.03692	0.13023	0.62779	0.24085	0.44116
2	0.85787	0.84618	0.00942	0.26121	0.32474	0.13990	0.39709	0.02646	0.01777	0.29374	0.18160	0.00038	0.04319	0.29874	0.41590	0.03269	0.10988	0.48600	0.18480	0.41459
2	0.43025	0.72905	0.01820	0.18499	0.18038	0.13894	0.34212	0.00378	0.01331	0.21260	0.14225	0.00024	0.03636	0.30191	0.27001	0.02338	0.11586	0.24568	0.18047	0.63268
…	…	…	…	…	…	…	…	…	…	…	…	…	…	…	…	…	…	…	…	…

**Table 8:** The distribution of the optimal parameter solution sets obtained by the nine combinations of the objective functions in the 5-segment position interval.

ID	Number					
	Excellent	Good	Medium	Pass	Poor	Sum
1	0	3	2	0	1	6
2	6	19	14	18	18	75
3	2	0	0	0	0	2
4	0	1	0	1	0	2
5	0	1	1	0	1	3
6	0	6	3	2	1	12
7	6	7	14	11	6	44
8	2	3	1	2	3	11
9	1	2	0	2	5	10
Sum	17	42	35	36	35	165

**Table 9:** The relative closeness values and the related combination ID of objective functions of the top 17 Pareto optimal solutions.

Rank	ID	Closeness
1	**2**	**0** **.9519** **6** ^*∗*^
2	**2**	0.95024
3	**2**	0.94885
4	**2**	0.94530
5	8	0.94344
6	**2**	0.93781
7	7	0.93676
8	8	0.93549
9	7	0.93484
10	9	0.92266
11	7	0.92189
12	3	0.92087
13	7	0.92072
14	7	0.91376
15	3	0.91131
16	**2**	0.91125
17	7	0.90254

**Table 10:** The calibrated model parameters related to the optimal solution for 9 combinations of objective functions.

ID	*K*	IMP	*B*	WUM	WLM	WDM	*C*	FC	KKG	Kr	Closeness	Rank	Score
2	0.22421	0.26762	0.60919	16.46257	92.43665	76.03592	0.08534	14.96132	0.99000	5.04023	0.95196^*∗*^	1	Excellent
8	0.22900	0.27194	1.00000	30.00000	100.00000	50.00000	0.06289	50.00000	0.99000	6.00000	0.94344	5	Excellent
7	0.25460	0.27288	0.58573	20.55312	50.00000	155.84605	0.30000	43.06852	0.99000	4.68565	0.93676	7	Excellent
9	0.22756	0.28869	0.20751	17.31577	99.45902	188.86870	0.30000	27.70888	0.99000	5.20626	0.92266	10	Excellent
3	0.26536	0.33720	0.28965	5.00000	86.74806	135.53369	0.03572	14.86729	0.99000	6.00000	0.92087	12	Excellent
1	0.14949	0.30469	0.20173	30.00000	50.00000	169.39066	0.06320	7.27659	0.99000	5.31799	0.89091	23	Good
6	0.26275	0.32561	0.47788	5.00000	50.00000	194.30614	0.22315	26.12875	0.99000	4.42370	0.88438	30	Good
4	0.40476	0.28015	0.57063	17.51399	64.47000	146.24538	0.04138	46.78419	0.99000	5.62186	0.82841	47	Good
5	0.34880	0.31021	0.38449	29.64771	76.45051	158.22721	0.07400	30.96984	0.99000	5.79101	0.80506	55	Good

**Table 11:** The 19 objective function values for optimal parameter solution related to the 9 combinations of objective functions.

ID	Ranked	NSE	LNNSE	*E*_RMOV_	*E*_RMS_	*E*_RMS_*H*1_	*E*_RMS_*L*1_	*E*_MSL_	*E*_*M*4_	*E*_MSD_	*R*_*E*_	*E*_TRMS_	*E*_ROC_	*E*_SFDC_	*R* ^2^	*P*_BIAS_	*E*_MSR_	*E*_RMS_*L*2_	*E*_RMS_*H*2_	*E*_MA_*L*_
2	1	0.224411	0.324177	0.146383	0.133598	0.211239	0.068281	0.152127	0.002154	0.013908	0.00209	0.098236	1.89*E* − 06	0.033992	0.222801	0.002085	0.010652	0.051542	0.254429	0.069254
8	5	0.228545	0.331114	0.139374	0.134823	0.211154	0.084607	0.155383	0.002314	0.01436	0.028368	0.098456	2.64*E* − 05	0.03239	0.227214	0.029196	0.010726	0.068202	0.262209	0.070093
7	7	0.231788	0.360839	0.109157	0.135776	0.201755	0.081997	0.169332	0.00198	0.014118	0.035732	0.100986	3.35*E* − 05	0.034186	0.226961	0.037056	0.011512	0.063049	0.245986	0.075922
9	10	0.220835	0.334022	0.129287	0.13253	0.219724	0.065059	0.156747	0.002163	0.01439	0.073192	0.097733	6.17*E* − 05	0.033235	0.213095	0.0682	0.010745	0.048941	0.258747	0.072094
3	12	0.227931	0.340734	0.125054	0.134642	0.195793	0.043117	0.159897	0.002305	0.014568	0.078854	0.099001	6.61*E* − 05	0.034632	0.201841	0.073091	0.010968	0.030586	0.254526	0.062349
1	23	0.326184	0.305082	0.161983	0.161068	0.216817	0.059866	0.143166	0.007955	0.014908	0.060137	0.109945	5.79*E* − 05	0.039857	0.205292	0.063985	0.011787	0.039573	0.346855	0.059945
6	30	0.284305	0.379421	0.103517	0.150373	0.192809	0.085178	0.178052	0.003634	0.015273	0.093578	0.109588	9.34*E* − 05	0.037341	0.221026	0.103239	0.012906	0.05772	0.276669	0.074355
4	47	0.2723	0.387395	0.100926	0.147164	0.269888	0.037762	0.181794	0.003725	0.01459	0.185758	0.107868	1.42*E* − 04	0.031669	0.232681	0.156658	0.01284	0.026698	0.303201	0.064164
5	55	0.259273	0.442913	0.059219	0.143601	0.246645	0.039434	0.207847	0.002893	0.014525	0.212467	0.106972	1.59*E* − 04	0.032653	0.211087	0.175235	0.013369	0.027712	0.279233	0.071378

**Table 12:** Performance comparison of combinations 2 and 7 of objective functions for the parameter optimization problem.

ID	GD		SP		IGD		HV		Time (second)	
	Mean	SD	Mean	SD	Mean	SD	Mean	SD	Mean	SD
2	**0.290118**	**0.00223**	**0.03885**	**0.08202**	**0.294291**	0.09878	**0.423085**	**0.04389**	6.617957	0.13140
7	0.290127	0.01828	0.09635	0.09766	0.294298	**0.01061**	0.419002	0.05696	**4.154882**	**0.08859**

## Data Availability

The observed data for the Xinanjiang model can be downloaded from the website of Scienific Data Center of Cold and Arid Regions: http://westdc.westgis.ac.cn.

## Additional Points

*Featured Application*. The proper selection of objective functions' combination for multiobjective problem has an important impact on the model's accuracy and efficiency. The technique for order preference by similarity to ideal solution (TOPSIS) based on the entropy theory is adapted to sort the Pareto optimal solutions to compare these combinations of objective functions. The method is applied in the multiobjective problem of hydrological models' parameter optimization and is proved to provide more comprehensive and impersonal decision support for hydrological forecasting.
